# Lightweight Multi-Scale Framework for Human Pose and Action Classification

**DOI:** 10.3390/s26041102

**Published:** 2026-02-08

**Authors:** Alireza Saber, Mohammad-Mehdi Hosseini, Amirreza Fateh, Mansoor Fateh, Vahid Abolghasemi

**Affiliations:** 1 Faculty of Computer Engineering, Shahrood University of Technology, Shahrood 36199-95161, Iran; alireza7448@gmail.com; 2 Department of Computer Engineering, Sha.C., Islamic Azad University, Shahrood 43189-36199, Iran; hosseini_mm@iau.ac.ir; 3 School of Computer Engineering, Iran University of Science and Technology (IUST), Tehran 13114-16846, Iran; amirreza_fateh@comp.iust.ac.ir; 4 School of Computer Science and Electronic Engineering, University of Essex, Colchester CO4 3SQ, UK

**Keywords:** lightweight, multi-scale, human pose, classification

## Abstract

Human pose classification, along with related tasks such as action recognition, is a crucial area in deep learning due to its wide range of applications in assisting human activities. Despite significant progress, it remains a challenging problem because of high inter-class similarity, dataset noise, and the large variability in human poses. In this paper, we propose a lightweight yet highly effective modular attention-based architecture for human pose classification, built upon a Swin Transformer backbone for robust multi-scale feature extraction. The proposed design integrates the Spatial Attention module, the Context-Aware Channel Attention Module, and a novel Dual Weighted Cross Attention module, enabling effective fusion of spatial and channel-wise cues. Additionally, explainable AI techniques are employed to improve the reliability and interpretability of the model. We train and evaluate our approach on two distinct datasets: Yoga-82 (in both main-class and subclass configurations) and Stanford 40 Actions. Experimental results show that our model outperforms state-of-the-art baselines across accuracy, precision, recall, F1-score, and mean average precision, while maintaining an extremely low parameter count of only 0.79 million. Specifically, our method achieves accuracies of 90.40% and 87.44% for the 6-class and 20-class Yoga-82 configurations, respectively, and 94.28% for the Stanford 40 Actions dataset.

## 1. Introduction

Human pose and action classification are fundamental tasks in computer vision and deep learning, aiming to identify a person’s posture or activity in still images or video sequences [1,2]. These tasks have broad applications in areas such as smart fitness, healthcare monitoring, rehabilitation, sports analysis, surveillance, and human–computer interaction [3,4,5]. A specialized subset of this field is yoga pose classification, which focuses on identifying specific postures practiced in yoga. Unlike general activity recognition, yoga pose classification requires a fine-grained understanding of body-part alignment and subtle differences in joint orientation. This can vary significantly even between visually similar poses [6]. It is particularly valuable in domains such as virtual fitness training [7], posture correction [8], and interactive health systems [9,10,11].

Typically, pose and action classification pipelines begin by extracting visual features from input images or sequences. This is followed by modeling the spatial arrangement of body parts and contextual dependencies across different regions. These features are then mapped to a set of predefined classes [12]. In traditional systems, handcrafted features or pose estimation outputs were commonly used [13]. However, these approaches often suffered from sensitivity to noise, occlusions, and variations in lighting or viewpoint [14].

The introduction of deep learning has brought significant progress to this field [15]. Convolutional neural networks (CNNs) initially dominated pose and action recognition by learning hierarchical visual representations directly from images [6,16]. More recent advancements, such as Transformer-based approaches [17,18] and Swin Transformers [19], have helped models to capture long-range dependencies, hierarchical features, and enhanced relationships among body parts. Several works have successfully applied these architectures to yoga pose recognition, sports activity classification, and human action detection, achieving remarkable improvements over earlier CNN-based methods [20]. In parallel, attention mechanisms have been widely incorporated into deep learning frameworks to highlight the most informative spatial and temporal features. These features allowing models to better discriminate between subtle variations in human poses [21,22]. By focusing computational resources on key regions or feature channels, attention-based methods improve recognition accuracy while maintaining efficiency.

Despite all these advancements, important challenges remain unresolved. The high similarity between certain classes, large intra-class variation caused by differences in individual execution of poses or actions, and dataset issues such as imbalance or noise still limit the accuracy and generalizability of existing models. Furthermore, many state-of-the-art methods rely on heavy architectures with high computational costs, making them unsuitable for real-time or resource-constrained environments. Another critical issue is the lack of interpretability, as most systems do not provide meaningful insights into their decision-making process, which reduces trust in practical applications.

To address these challenges, we propose a novel multi-scale deep learning architecture based on a modular attention design and a Swin Transformer backbone. Our model is trained and evaluated across multiple yoga pose datasets to demonstrate its robustness in handling pose variations and class imbalances. Specifically, our architecture begins by extracting hierarchical features from four stages of a pretrained Swin Transformer, which is kept frozen to retain its robust visual representations and prevent overfitting. This also significantly reduces training time and computational cost. The resulting multi-scale feature maps are unified in resolution and channel dimension through our feature fusion module. Furthermore, to enhance pose representation, we apply an Enhanced Spatial Attention (SPA) module and a Context-Aware Channel Attention Module (CCAM) to concatenated feature maps at different semantic levels. After that, a Dual Weighted Cross-Attention (DWCA) module is introduced to model relationships over spatially and channel-wise attended features. Finally, the output feature map of the DWCA module goes through global average pooling and passes through a compact, expressive classifier head.

Overall, our model design enables effective representation of subtle pose differences, adapts well to intra-class variation, and offers interpretable, attention-driven outputs with minimal latency, making it well suited for yoga pose classification applications. The contributions of our work are summarized as follows:

- We propose a novel attention-based deep learning architecture that integrates multi-scale hierarchical features with both spatial and channel attention mechanisms for effective yoga pose classification.
- Achieves state-of-the-art accuracy on Yoga-82 and Stanford 40 Actions with an extremely low parameter count (0.79 million), making it suitable for real-time applications.
- Utilizes and proposes modified modules such as SPA and CCAM.
- Introduces learnable gating to balance cross-attended and raw fused features, improving fine-grained pose discrimination.
- Employs explainable AI techniques to increase the interpretability and trustworthiness of our model.
- Accurately distinguishes subtle intra-class variations, which is crucial for yoga pose classification.

The rest of this paper is organized as follows. In Section 2, we review some of the related works. Section 3 presents the details of our proposed architecture and attention modules. Section 4 outlines the experimental setup and dataset configuration, followed by an evaluation of performance and qualitative analysis. Finally, Section 5 concludes the paper and discusses future directions.

## 2. Related Works

Early approaches to human pose and action classification relied primarily on handcrafted features and pose keypoints extracted through human pose estimation techniques [12,13]. While these methods provided foundational insights, they were highly sensitive to environmental variations, such as changes in lighting, occlusions, or errors in keypoint localization [14]. As a result, their ability to generalize across diverse and complex human poses was limited. A review of the existing literature shows that a variety of strategies have been introduced for automated human pose detection, which can be broadly categorized into three main groups: transformer-based methods, transfer learning approaches, and attention mechanisms.

### 2.1. Transformers

Transformers have recently gained significant attention in human pose and action classification due to their ability to capture long-range dependencies across spatial and temporal dimensions. Unlike CNNs, which primarily rely on local receptive fields, Transformers provide a global context that is particularly beneficial for modeling complex body configurations. For instance, Hassanin et al. [23] introduce two novel modules, Cross-Joint Interaction and Cross-Frame Interaction, which explicitly encode both local and global dependencies between body joints. This design enables the network to capture subtle inter-frame variations and fine-grained joint relationships, which are critical for precise pose understanding. Hongwei Zheng et al. [24] leveraged a Transformer-based hierarchical autoregressive modeling scheme to generate dense 2D poses from sparse skeleton inputs, addressing occlusion challenges in 2D-to-3D human pose estimation. By tokenizing skeletons into multi-scale hierarchical representations, this approach strengthens spatial dependencies through Skeleton-aware Alignment, which aligns closely with the ability of Transformers to model long-range relationships.

### 2.2. Transfer Learning

Transfer learning has known as a powerful strategy to improve model performance in scenarios with limited labeled data by leveraging knowledge from related source domains. It enables models to generalize better across tasks or datasets, making it particularly useful in applications such as human activity recognition and multi-task sensor-based learning. İşgüder et al. [25] demonstrated the use of Federated Transfer Learning to leverage motion sensor data for multiple tasks, including human activity recognition and device position identification. By applying transfer learning across ten smaller datasets in the OpenHAR framework, this approach enables task-specific and personalized models without centralized data aggregation. Thukral et al. [26] introduced Cross-Domain HAR, a transfer learning framework for sensor-based human activity recognition that leverages publicly available datasets to overcome limited labeled data in target domains. By employing a teacher–student self-training paradigm, it effectively bridges differences between source and target domains, including variations in sensor placement and activity types. Akash et al. [27] applied transfer learning with VGG-16, ResNet, and DenseNet-121, combined with Neural Architecture Search, to improve classification accuracy on the Yoga-82 dataset.

### 2.3. Attention Mechanism

Attention mechanisms have also been widely used in human pose classification to enhance feature learning and emphasize the most informative regions of the image. Xiongwei and Zifan Wang [28] proposed TCN-Attention-HAR, a recognition model that combines temporal convolutional networks with attention mechanisms to enhance human activity recognition from wearable sensor data. The attention module highlights key temporal features, allowing the model to focus on the most informative signals and improve recognition accuracy across benchmark datasets. Lei Zhang et al. [29] introduced a multi-channel hybrid deep learning framework that integrates convolutional, recurrent, and attention modules for sensor-based human activity recognition. The attention mechanism enables the model to emphasize the most informative spatial and temporal features, thereby improving recognition accuracy in multi-position sensor fusion scenarios. Weirong Sun et al. [30] enhanced action recognition by introducing a k-NN attention-based Video Vision Transformer (ViViT), which refines the standard self-attention mechanism. By focusing only on the most relevant tokens and discarding noisy or non-informative ones, the model reduces computational complexity while maintaining strong spatio-temporal modeling. Elaheh Dastbaravardeh et al. [31] introduced a CNN-based framework enhanced with channel attention mechanisms (CAMs) and autoencoders for action recognition in low-resolution and small-size videos. The CAMs allow the network to focus on the most discriminative feature channels, improving recognition accuracy while keeping computational costs low.

Overall, recent advances in human pose and action recognition highlight the effectiveness of integrating Transformers, transfer learning strategies, and attention mechanisms into deep learning frameworks. Transformers contribute by modeling long-range spatial dependencies and capturing fine-grained joint relationships, while transfer learning enables robust generalization across domains with limited labeled data. Attention mechanisms further enhance recognition by emphasizing the most discriminative features in both spatial and temporal dimensions, improving efficiency and accuracy in complex or low-quality data scenarios. Despite these advances, challenges remain in reducing computational overhead, handling occlusions, and ensuring generalization across diverse conditions, which are essential for the reliable deployment of human pose recognition applications.

## 3. Proposed Method

### 3.1. Overview

**Figure 1:**
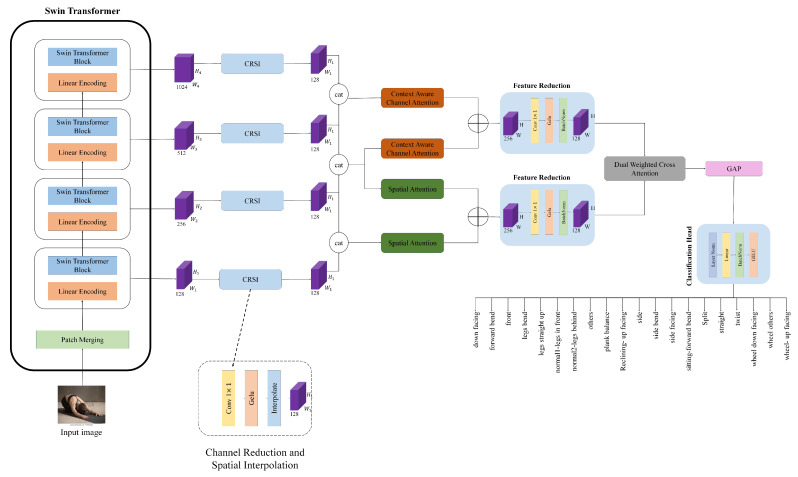
An overview of the architecture of the proposed method. We show the predicted labels from Yoga-82 dataset as an example; however, the proposed method is the same for both datasets.

In this study, we propose a deep learning architecture for robust image classification across diverse yoga pose datasets. The overall architecture of our model is illustrated in Figure 1. Our method integrates several key components to enhance classification performance. First, we employ a pretrained Swin Transformer as the backbone to extract hierarchical, multi-scale feature maps from four distinct stages. These features are processed through dimension reduction layers to ensure computational efficiency. Subsequently, we implement a sophisticated feature enhancement pipeline, incorporating Enhanced SPA, CCAM, and DWCA modules to emphasize critical spatial and channel-wise information. The resulting feature representations are merged, globally pooled, and fed into a lightweight classifier composed of normalization, linear layers, and dropout for robust predictions. This process is also shown in the pseudocode in Algorithm 1 for better understanding to our architecture.

### 3.2. Backbone

The backbone of our model is built upon the Swin Transformer. Specifically, we used the swin_b (base) variant with a patch size of 4, a window size of 7, and an input size of 224 × 224. The Swin Transformer employs a hierarchical architecture with shifted window-based self-attention, enabling it to capture both local and global contextual information efficiently. This model generates four feature maps at different scales, which are critical for our multi-scale feature fusion strategy. By freezing the backbone parameters, we preserve the learned representations while reducing computational overhead during training, ensuring that the model focuses on fine-tuning the subsequent attention and classification modules. The selection of the Swin Transformer as our backbone is motivated by several key advantages. First, its hierarchical structure and window-based attention mechanism provide a balance between computational efficiency and the ability to model long-range dependencies, making it ideal for processing high-resolution images such as those used in our study. Second, the pretrained weights of the Swin Transformer, derived from extensive datasets like ImageNet, offer a strong initialization point, enhancing the model’s ability to generalize across diverse classification tasks, specifically for human images as considered in our paper. Additionally, its feature extraction capability, as evidenced by the multi-scale outputs, aligns seamlessly with our architecture’s need for rich, multi-level feature representations. These characteristics make the Swin Transformer a superior choice over traditional convolutional neural networks or other transformer-based models for our application, ensuring robust performance in complex image classification scenarios.
**Algorithm 1:** Overall explanation of our framework.
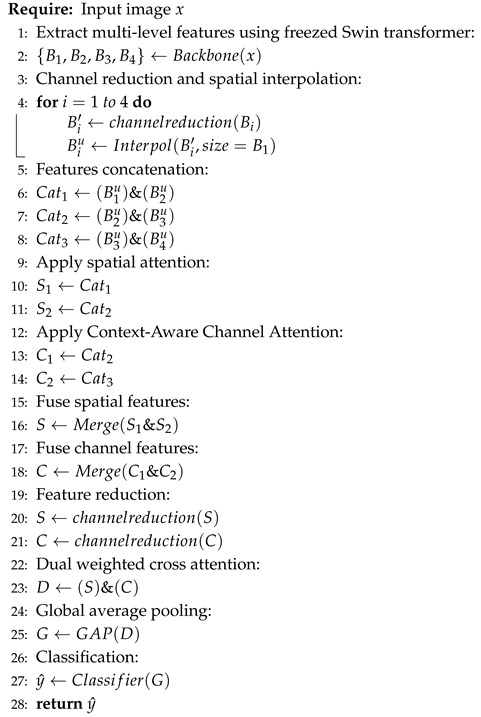


### 3.3. Multi-Scale Feature Extraction

Our proposed method begins with feature extraction using the Swin Transformer backbone, which generates four feature maps ($B_{1}$, $B_{2}$, $B_{3}$, $B_{4}$), increasing channel dimensions from 128 at block 1 ($B_{1}$) to 1024 at block 4 ($B_{4}$) and reducing spatial dimensions from H = W = 56 in block 1 ($B_{1}$) to H = W = 7 in block 4 ($B_{4}$). To standardize feature representation and facilitate effective multi-level fusion, we pass each of these feature maps through a channel reduction and spatial interpolation (CRSI) module. This module first changes the number of channels to a fixed intermediate channel size using a 1×1 convolutional layer (denoted as $B_{i}^{\prime }$), where i∈1,2,3,4, and then upsamples them using bilinear interpolation to match the spatial resolution of the first block, denoted as $B_{i}^{u}$. These operations are implemented in Equations (1) and (2):

(1) $$B_{i}^{\prime }={\mathrm{Conv}}_{1\times 1}\left(B_{i}\right)$$

(2) $$B_{i}^{u}=\mathrm{Interpol}(B_{i}^{\prime };size=\left(H_{1},W_{1}\right))$$

where Interpol represents the interpolation operation.

### 3.4. Spatial Attention

**Figure 2:**
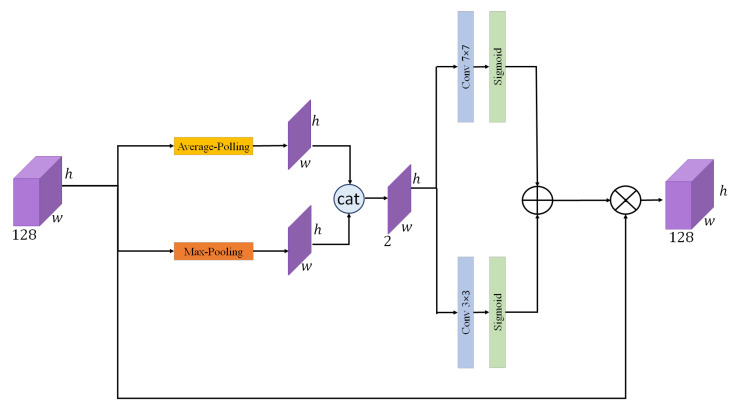
The illustration of the Spatial Attention module.

To emphasize informative regions within each feature map, we propose an SPA. As shown in Figure 2, this module augments traditional attention by leveraging multi-scale spatial context, enabling the model to better focus on semantically salient areas such as limbs, alignment, and body contours in yoga poses. To implement this spatial attention, firstly, we utilized both average pooling and max pooling, followed by concatenation to capture complementary context from the input feature map, denoted as (x), with (c, h, w) dimensions. The implementation of this is shown in Equation (3).

(3) $$F_{cap}\left(x\right)=Concat\left(avg\left(x\right),max\left(x\right)\right)$$

where avg(x) and max(x) represent average pooling and max pooling, respectively. $F_{cap}$ values have been assigned to two different convolutional branches with different kernel sizes, denoted as $F_{sa1}$ and $F_{sa2}$. In our SPA, we used two different kernels with size of 7 × 7 and 3 × 3, which is illustrated in Equations (4) and (5). Also, each convolution layer in our SPA includes a batch normalization followed by a sigmoid activation function.

(4) $$F_{sa1}=\sigma \left(BN\left(Conv_{7\times 7}\left(F_{cap}\left(x\right)\right)\right)\right)$$

(5) $$F_{sa2}=\sigma \left(BN\left(Conv_{3\times 3}\left(F_{cap}\left(x\right)\right)\right)\right)$$

where σ represents the sigmoid function and BN represents batch normalization. After these steps, we summed up the $F_{sa1}$ and $F_{sa2}$ via an element-wise addition, followed by an attention multiplication with the input feature map, which is shown in Equation (6):

(6) $$F_{spa}=\left(x\right)\times \left(F_{sa1}+F_{sa2}\right)$$

### 3.5. Context-Aware Channel Attention

**Figure 3:**
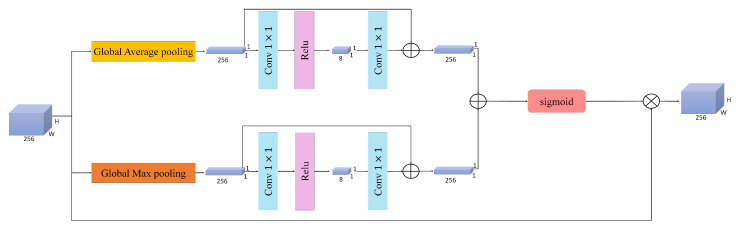
The architecture of the Context-Aware Channel Attention.

To enhance the discriminative power of the learned features, we incorporate a CCAM into our architecture. The illustration of this module is shown in Figure 3. This module is designed to emphasize the most informative channels by adaptively weighting them based on global contextual cues. It captures both global average pooling and global max pooling from the input feature map, enabling the model to understand the broader context of the image. Each of these two pooled features is then passed through a lightweight multi-layer perceptron (MLP), which is denoted as $F_{mlp}$. As shown in Equation (7), this MLP consists of two convolutional layers: the first reduces the number of channels to one-sixteenth of the original with a 1×1 convolution, followed by a ReLU activation, while the second convolution, with 1×1 kernel size, restores the channel dimension to match the dimension of the feature map before the MLP.

(7) $$F_{mlp}=Conv_{1\times 1}(Relu\left(Conv_{1\times 1}\right))$$

As described earlier, both the global average pooled and global max pooled descriptors are passed through the MLP to produce two attention maps, denoted as $F_{gap}$ and $F_{gmp}$. Then, in order to avoid losing spatial features from the original input, a sum up like residual connection in resnet has been added between the output of each MLP with the output feature map of GAP(x) or GMP(x), as expressed in Equations (8) and (9):

(8) $$F_{gap}=F_{mlp}\left(GAP\left(x\right)\right)+GAP\left(x\right)$$

(9) $$F_{gmp}=F_{mlp}\left(GMP\left(x\right)\right)+GMP\left(x\right)$$

where GAP and GMP represent global average pooling and global max pooling, respectively. Finally, the combined output of both $F_{gap}$ and $F_{gmp}$ is passed through a sigmoid function and then multiplied with the input feature map (x) to produce the final channel attention map, as shown in Equation (10):

(10) $$F_{ccam}=\left(x\right)\times \left(\sigma \left(F_{gap}+F_{gmp}\right)\right)$$

where σ represents the sigmoid activation function. This mechanism allows the model to selectively focus on more relevant features, improving classification performance in tasks such as yoga pose recognition.

### 3.6. Dual Weighted Cross Attention

**Figure 4:**
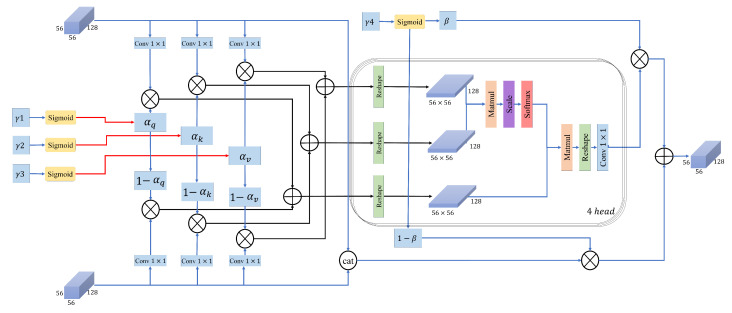
The architecture of the proposed Dual Weighted Cross Attention.

A novel module is proposed to enhance the interaction between different feature representations. The architecture of this module is illustrated in Figure 4. Unlike conventional cross-attention modules that apply fixed query (Q), key (K), and value (V) projections, our DWCA introduces learnable gating parameters that dynamically control the contribution of each input stream to the combined Q, K, and V representations. This approach was adopted due to the importance of both input feature maps, which are the outputs of Spatial Attention ($F_{spa}$) and CCAM ($F_{ccam}$). To the best of our knowledge, existing cross-attention or gated-attention approaches typically apply gating at the feature level or attention output, rather than explicitly controlling the participation of multiple inputs at the level of individual Q/K/V projections. This design allows the model to flexibly emphasize one feature stream or jointly exploit both, depending on the input characteristics and task requirements. In addition to improving feature interaction, this dynamic behavior can implicitly reduce unnecessary computation when one input stream is considered less informative, making DWCA particularly suitable for resource-constrained settings. Three separate 1×1 convolutions are applied to each input feature map. The resulting features are then scaled using learnable gates via element-wise multiplication. Finally, the gated outputs from both branches are combined through element-wise addition to compute the final query (Q), key (K), and value (V) representations. These operations are shown in Equations (11)–(13).

(11) $$Q=\left(Conv_{1\times 1}\left(F_{spa}\right)\times \alpha_{q}\right)+\left(Conv_{1\times 1}\left(F_{ccam}\right)\times \left(1-\alpha_{q}\right)\right)$$

(12) $$K=\left(Conv_{1\times 1}\left(F_{spa}\right)\times \alpha_{k}\right)+\left(Conv_{1\times 1}\left(F_{ccam}\right)\times \left(1-\alpha_{k}\right)\right)$$

(13) $$V=\left(Conv_{1\times 1}\left(F_{spa}\right)\times \alpha_{v}\right)+\left(Conv_{1\times 1}\left(F_{ccam}\right)\times \left(1-\alpha_{v}\right)\right)$$

where α represents the learnable parameter of our designed module. The parameter α is initialized to 0.5, corresponding to an equal contribution from both inputs at the beginning of training, and is dynamically updated through backpropagation. No additional normalization layer is applied within the DWCA module, because the gating parameters are constrained through a sigmoid activation, implicitly bounding the fusion weights and mitigating potential scale imbalance. Furthermore, we have batch normalization and layer norms in our classification head and adding more could lead to over-normalization. The resulting Q, K, and V tensors are then reshaped to a size of (C, H × W), where C is the number of channels and H × W is the flattened spatial dimension. Specifically, each of these tensors is split into h attention heads along the channel dimension, where the dimensionality of each head is d = C/h. For each head, attention is computed individually, as detailed in Equation (14):

(14) $$Attention\left(Q,V,T\right)=Softmax\left(\frac{Q^{t}.K^{t}}{\sqrt{d}}\right)V^{t}$$

where $Q^{t}$, $K^{t}$, and $V^{t}$ represents the reshaped tensors of query, key, and value, respectively. In addition to input-level blending, DWCA incorporates a learnable gating mechanism at the output stage to dynamically decide between attention-enhanced features and the original fused representations. After computing the attention-weighted representations, the output is projected back to the original feature dimension. Simultaneously, the two input feature maps are concatenated and reduced to the baseline feature maps dimension, which is denoted as $F_{concat}$. This operation and final output are shown in Equations (15) and (16).

(15) $$F_{concat}=Concat\left(F_{spa},F_{ccam}\right)$$

(16) $$DWCA=\left(\beta \times Attention\right)+(\left(1-\beta \right)\times F_{cat})$$

where β is the learnable gating parameter. This β is the same as α, set as 0.5 initially and changed during the learning procedure. This gating mechanism allows DWCA to adaptively emphasize either the cross-attended features or the raw fused features, depending on which provides more informative cues for the task. Overall, DWCA offers a flexible mechanism for feature interaction, enabling more precise reasoning over pose-specific representations in complex visual scenes.

### 3.7. Loss Function

For training our model, we employ the Cross-Entropy Loss, a widely used objective function for multi-class classification tasks. Cross-entropy measures the discrepancy between the predicted probability distribution and the ground-truth labels, penalizing incorrect predictions with higher loss values. This formulation encourages the model to assign higher confidence to the correct class while reducing the probability of misclassification. The implementation of this function is shown in Equation (17)

(17) $$F_{loss}=-\frac{1}{N}\sum_{i=1}^{N}\left[y_{i}\cdot \log\left({\hat{y}}_{i}\right)+\left(1-y_{i}\right)\cdot \log\left(1-{\hat{y}}_{i}\right)\right]$$

where *N* is the number of classes, $y_{i}$ represents the label, and ${\hat{y}}_{i}$ is the probability of positive class.

## 4. Experimental Result

### 4.1. Dataset

In this study, we utilized two different datasets to enhance the generalization capability of our model. One of these datasets is the Yoga-82 dataset [32], which contains approximately 16,800 images of yoga poses captured in various positions. These images are hierarchically organized into 6 main categories, which are further divided into 20 subcategories. Each of these subcategories is further split into finer subclasses, resulting in a total of 82 pose classes. The second dataset used in this study is the Stanford 40 Actions dataset [33]. As its name suggests, this dataset contains images of different actions categorized into 40 distinct classes. In total, the dataset comprises 9532 images.

#### 4.1.1. Yoga-82

The Yoga-82 dataset serves as the primary dataset for our work. To evaluate our model comprehensively on this dataset, we trained it in two separate phases: in the first phase, we focused on the 6 main classes, while in the second phase, we expanded the training to include all 20 subclasses of these 6 classes. One of the key challenges of this dataset is its high intra-class variability and inter-class similarity, as many yoga poses share subtle visual differences in posture and body orientation, making accurate classification difficult. Additionally, the dataset contains some noisy or irrelevant samples, such as blank images or images with only text, which can negatively affect model training. To mitigate this issue, we carefully filtered out such incorrect samples before training to ensure cleaner and more reliable data. For fair evaluation, we followed the original Yoga-82 dataset protocol and split the data into training and testing sets in the same manner as reported in the base paper. The only difference is that 10% of the training data is further separated and used as a validation set.

#### 4.1.2. Stanford 40 Actions

The Stanford 40 Actions dataset is employed as the secondary dataset in our study to evaluate and enhance the generalization capability of the proposed model. This dataset presents significant challenges due to the high variability in human poses, diverse backgrounds, and frequent occlusions, which make action recognition more complex. To maintain consistency and fairness, we follow the same train–test split strategy as outlined in the original dataset paper.

#### 4.1.3. Data Augmentation

To improve the robustness and generalization ability of our model, we applied a series of data augmentation techniques during training. Specifically, we used random resized cropping to ensure the model learns scale-invariant features by randomly cropping and resizing the images to 224 × 224. A random vertical flip was included to simulate variations in pose orientation, which is particularly beneficial for yoga poses and action recognition tasks. We also applied color jittering with controlled adjustments to brightness, contrast, saturation, and hue, which helps increase data diversity. Finally, all images were normalized using the ImageNet mean and standard deviation to match the pretraining statistics of the Swin Transformer backbone, enabling more effective transfer learning.

### 4.2. Experimental Settings

All experiments were conducted using Python 3.11.13 with PyTorch 2.6.0 as the primary deep learning framework. We trained our model with a batch size of 16 and an initial learning rate of $1\times 10^{-3}$, optimized using the Adam optimizer. The training process was carried out for 60 iterations (epochs). To ensure reliability and minimize variance, the training process was repeated five times, and the average performance across runs is reported as the final result. All experiments were conducted on Kaggle servers equipped with an NVIDIA Tesla P100 GPU (16 GB VRAM), 13 GB of system RAM, and an Intel Xeon 2.3 GHz CPU.

### 4.3. Training and Validation Analysis

**Figure 5:**
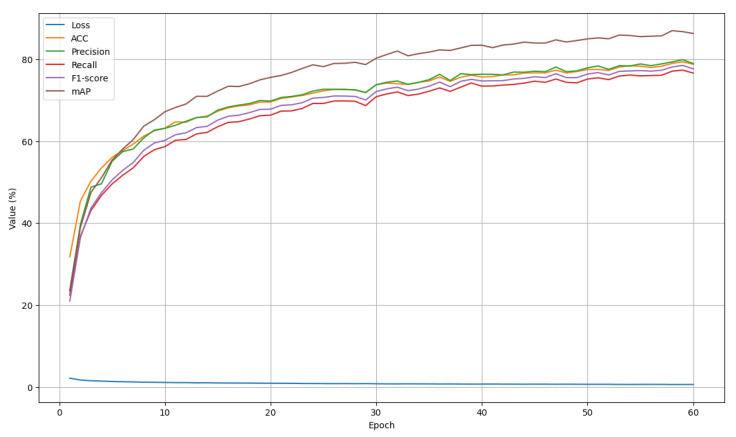
The diagram of training performance metrics across 60 epochs.

**Figure 6:**
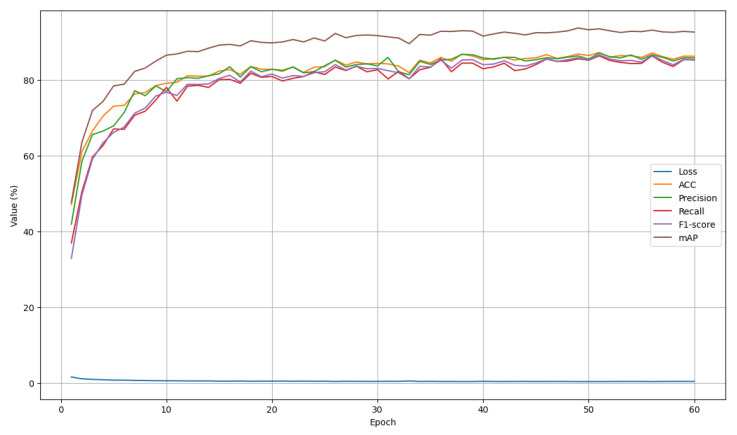
The diagram of validation performance metrics across 60 epochs.

Figure 5 and Figure 6 present the training and validation diagrams of loss, accuracy, precision, recall, F1-score, and MAP over 60 epochs. As shown in Figure 5, the training loss decreases rapidly during the early epochs and gradually converges, indicating effective optimization and stable learning behavior. Simultaneously, all training performance metrics exhibit a consistent upward trend, with pronounced improvements in the initial stages followed by smoother refinements in later epochs.

Figure 6 presents the corresponding validation curves, which closely follow the training trends across all metrics. After an initial rapid improvement phase, the validation performance stabilizes with minor fluctuations, suggesting that the model generalizes well to unseen data. Notably, the gap between training and validation curves remains limited throughout the training process, indicating the absence of significant overfitting. The steady behavior of MAP alongside classification metrics further confirms the robustness and consistency of the learned representations.

### 4.4. Evaluation Metrics

To comprehensively assess the performance of our model, we employed accuracy, precision, recall, F1-score, and mean average precision (MAP) as evaluation metrics. In this study, we used accuracy, precision, recall and F1-score, for comparing with other state of the arts in Section 4 for both datasets and ablation study. Moreover, MAP was adopted as an additional metric to compare our work with prior studies on the Stanford 40 Actions dataset. Accuracy provides an overall measure of correctly classified samples, while precision and recall evaluate the model’s ability to minimize false positives and false negatives, respectively. The F1-score, which is the harmonic mean of precision and recall, offers a balanced evaluation in cases of class imbalance. Furthermore, MAP was used to capture the model’s performance across all classes by considering the average precision at multiple recall thresholds, providing a more robust metric for multi-class classification.

(18) $$Accuracy=\frac{TP+TN}{TP+TN+FP+FN}$$

(19) $$\mathrm{Precision}=\frac{TP}{TP+FP}$$

(20) $$Recall=\frac{TP}{TP+FN}$$

(21) $$F1-\mathrm{score}=2\times \frac{\mathrm{Precision}\times \mathrm{Recall}}{\mathrm{Precision}+\mathrm{Recall}}$$

(22) $$AP=\sum_{1}^{k}{\mathrm{Precision}}_{k}({\mathrm{Recall}}_{k}-{\mathrm{Recall}}_{k-1})$$

(23) $$MAP=\frac{1}{n}\sum_{i=1}^{n}AP_{i}$$

where True Positive (TP) represents the number of samples correctly predicted as belonging to a given class, while True Negative (TN) denotes the samples correctly identified as not belonging to that class. False Positive (FP) refers to the samples that are incorrectly predicted as belonging to a class when they do not, and False Negative (FN) denotes the samples that truly belong to a class but are misclassified. In Equation (22), the Average Precision (AP) is computed by summing the product of precision and the change in recall at each step (k) along the precision–recall curve. In Equation (23), N denotes the total number of classes, and the mean Average Precision (MAP) is calculated as the average of the AP values across all classes.

### 4.5. Comparison with State-of-the-Art Methods

Our proposed method demonstrates consistent improvements over existing approaches on both the Yoga-82 and Stanford 40 Actions datasets, as shown in Table 1 and Table 2. For a fair comparison, the reported results of baseline methods were taken directly from their original publications. We note that their training settings (e.g., number of epochs, input resolution, or data augmentation) may differ from ours; therefore, minor discrepancies may exist. On Yoga-82, our model achieves an accuracy of 87.44% (20-class) and 90.4% (6-class), outperforming Verma et al. [32] (84.42% and 87.2%) and Borthakur et al. [34] (85%). Notably, our F1-score (86.39% for 20-class, 89.73% for 6-class) significantly surpasses Borthakur et al.’s 71%, indicating a better balance between precision and recall. This improvement can be attributed to our method’s ability to capture fine-grained pose variations, whereas prior works rely on rigid feature extraction, leading to misclassifications in similar-looking poses. On the Stanford 40 Actions dataset, our method achieves competitive results with a MAP of 94.28%, outperforming most existing approaches except Multi-Attention Guided Network [35] (94.2%) and Body Structure Cues [36] (93.8%). The strong performance in accuracy (90.27%) and F1-score (89.3%) suggests that our lightweight design effectively retains discriminative power while reducing redundancy. This is particularly evident in recall (89.47%), where our model generalizes better to underrepresented action classes compared to methods like R*CNN [37] and ResNet-50 [38], which report no recall values. The superior performance of our method stems from three key factors: first, efficient feature fusion that preserves spatial and contextual information; second, a parameter-efficient architecture that avoids overfitting while maintaining discriminative capacity; and third, robustness to intra-class variations. While some high-capacity models achieve marginally higher MAP, our method strikes a better balance between accuracy and efficiency.

### 4.6. Ablation Study

In this section, we conducted several ablation studies to evaluate our design in different scenarios. For a fair comparison, all of the following ablations were evaluated on the same settings. For example, all were trained on the yoga-82 dataset with the same number of classes, the same augmentation, and the same number of epochs. First, as shown in Table 3, we investigated the effectiveness of choosing different methods as our backbone. Our experiments show that the Swin Transformer performs significantly better than other strategies with our proposed method.

To assess the effectiveness of each core component in our architecture, we conducted detailed ablation experiments, as shown in Table 4. Starting from a baseline model that uses only the Swin Transformer backbone, we progressively introduced our proposed modules: Enhanced SPA, CCAM, and DWCA. The addition of SPA improved accuracy by capturing fine-grained spatial cues, while CCAM contributed further by emphasizing informative channels based on global context. When both modules were applied together, performance improved more significantly, indicating the complementary nature of spatial and channel attentions. Finally, the integration of DWCA provided the most substantial boost, demonstrating its strength in blending and refining the attended features through gated cross-attention. With all modules enabled, our model achieved the best performance across all metrics, confirming that each module plays a crucial role in enhancing the discriminative capability of the network. In terms of our claim of being lightweight, we also computed real-time information, such as the number of parameters, FLOPs, and inference time for each module. As shown in Table 4, our model used only 0.79 million parameters with an inference time of 26.91 ms in the final version with all modules. Furthermore, these numbers increased gradually from the baseline, which used 0.14 million parameters and 20.07 ms for inference time. These ablations overall demonstrate that our model consists of several useful modules to achieve high performance while remaining lightweight and practical for real-time applications.

To further evaluate the design of our CRSI (channel reduction and spatial interpolation) module, we tested multiple configurations that varied the number of output channels (64, 128, 256) and target spatial resolutions (14 × 14, 28 × 28, 56 × 56). Results from this ablation, summarized in Table 5, show that increasing the spatial resolution leads to better performance, highlighting the importance of preserving finer spatial details for pose classification. Among all tested variants, the CRSI configuration with 128 channels and a 56 × 56 output resolution achieved the highest accuracy and F1-score, balancing richness of feature representation with manageable model complexity. These experiments demonstrate that both the spatial and channel configurations of the fusion stage play a critical role in downstream classification accuracy and validate our design choice for CRSI as a compact yet effective feature unification module.

We also conducted a focused ablation on the design of our Enhanced SPA module by comparing it against standard CBAM-style spatial attention mechanisms using different convolutional kernels. As shown in Table 6, employing only a 7 × 7 convolution or only a 3 × 3 convolution yields modest performance gains over the baseline. However, when both kernel sizes are combined, the accuracy and F1-score increase substantially to 86.87% and 85.84%, respectively. This confirms that fusing multi-scale receptive fields enables the network to better capture diverse spatial patterns across pose variations, thereby enriching the attention maps used in downstream processing.

Additionally, to investigate the contribution of context modeling in the channel attention mechanism, we compared our CCAM with a simpler, non-contextual variant. As detailed in Table 7, the standard channel attention using only a 1 × 1 convolution achieves lower performance, while the context-aware version with added 1 × 1 convolutions and a residual connection attains a notable improvement across all metrics. This boost, increasing the F1-score to 85.84%, demonstrates that capturing spatial context within each channel is crucial for enhancing discriminative focus. By incorporating local context and global semantics, CCAM allows the model to more effectively highlight informative channels across different layers.

We also provide an ablation to demonstrate the effectiveness of our proposed DWCA compared to other fusion strategies such as addition, concatenation, or standard cross-attention in terms of metric performance. As shown in Table 8, our proposed DWCA performs significantly better than the other strategies. We believe one reason for this improvement is that the learnable weights help our model better assign and select key, query, and value components, which is crucial for transformer-based cross-attention modules.

### 4.7. Confusion Matrix

**Figure 7:**
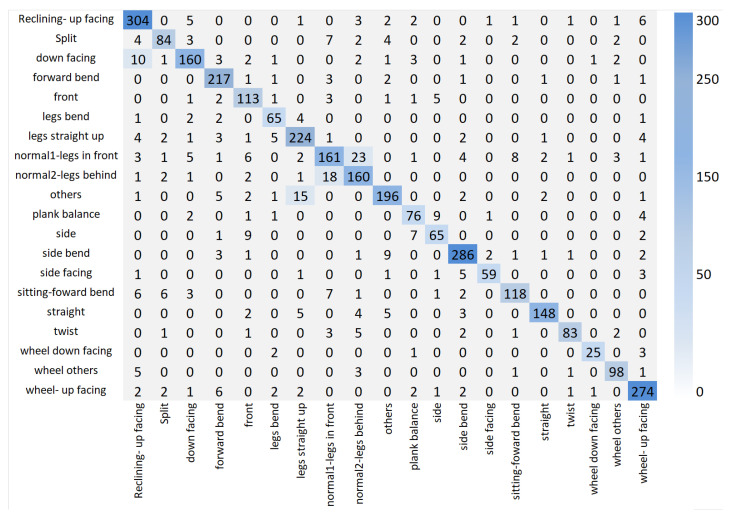
The confusion matrix illustration for the Yoga-82 dataset.

**Figure 8:**
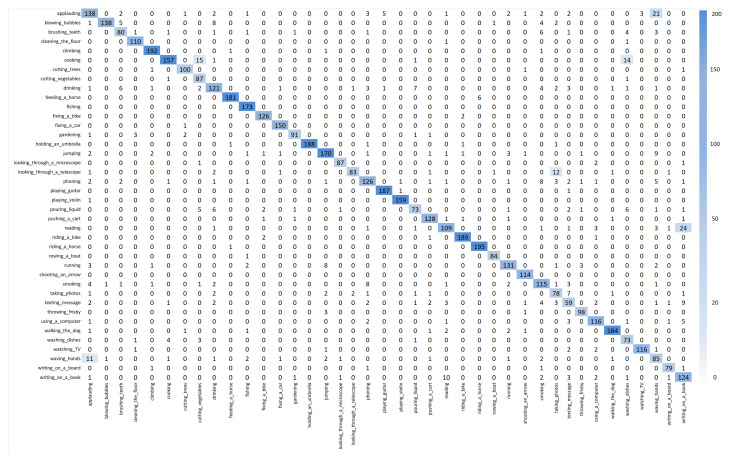
The confusion matrix illustration for Stanford 40 Actions.

A confusion matrix is a widely used evaluation tool in classification tasks, summarizing the relationship between predicted and actual labels. Correct predictions appear along the diagonal, while off-diagonal values indicate misclassifications. This visualization not only reflects overall accuracy but also reveals which specific classes are often confused, providing deeper insights into model performance. Figure 7 presents the confusion matrix for the Yoga-82 dataset using its 20 subclasses. The diagonal dominance across most classes, such as reclining-up facing, forward bend, and wheel-up facing, indicates that the model effectively distinguishes the majority of poses. However, certain categories, including split, legs bent, and normal1 legs in front, exhibit moderate confusion with visually similar poses that differ only in subtle limb orientations or camera angles. These misclassifications highlight the inherent difficulty of fine-grained yoga pose recognition, where high inter-class similarity and intra-class variation coexist. Figure 8 illustrates the confusion matrix for the Stanford 40 Actions dataset. Most action classes, such as applauding, climbing, and riding a bike, show strong diagonal performance, confirming the model’s robustness in recognizing diverse human activities. Nevertheless, actions with overlapping visual cues, such as phoning versus texting a message and waving hands versus applauding, occasionally result in misclassifications. This is largely due to shared contextual elements and similar upper-body movements, which can pose a challenge even for high-performing models. Despite these minor confusions, the overall results demonstrate consistently high performance across both datasets.

### 4.8. Gradient-Weighted Class Activation Mapping

Gradient-weighted Class Activation Mapping (Grad-CAM) is a widely used visualization technique for interpreting convolutional neural network (CNN) decisions. It generates class-discriminative localization maps by leveraging the gradients of a target class flowing into the final convolutional layer, thereby highlighting the image regions most relevant to the prediction.

**Figure 9:**
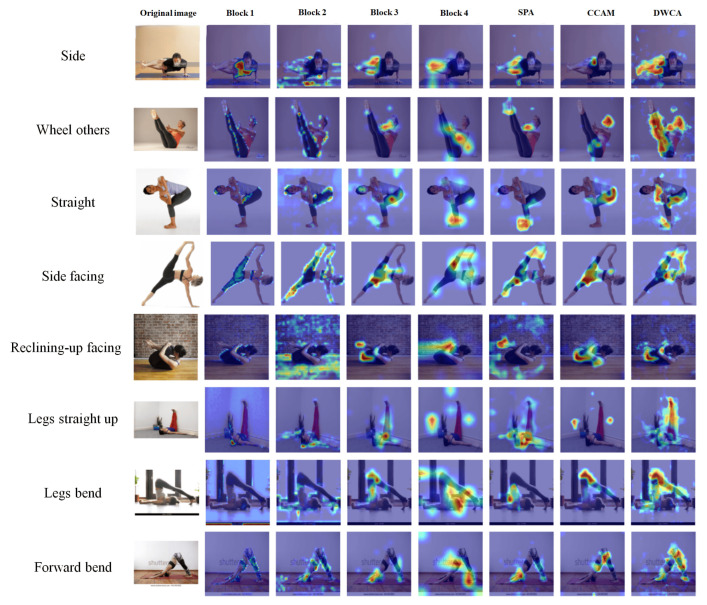
An illustration of Grad-CAM with the Yoga-82 dataset. Bolder colors such as red represents stronger attentions rather than blue and lighter colors.

In Figure 9, the Grad-CAM visualizations reveal the model’s attention patterns for complex yoga poses in the Yoga-82 dataset, where subtle limb positions can be critical for correct classification. Block 1 activations are usually spread over large body regions and parts of the background, making it difficult to distinguish fine posture details. Block 2 introduces some structural cues, but the highlighted areas remain broad and imprecise. By Block 3 and Block 4, the focus becomes more discriminative, centering on the main body configuration; for example, the raised legs in legs straight up or the arched back in wheel, among others. The attention modules then refine these maps further. SPA often emphasizes large connected body areas, CCAM pinpoints several critical joints or limbs, and DWCA frequently produces the sharpest focus on the most defining parts of the pose, such as the extended arms in side facing or the bent torso in forward bend. Across many examples, all three attention mechanisms surpass the localization quality of Block 4, with our model sometimes achieving particularly well-targeted focus in the DWCA stage, aligning closely with the most discriminative regions for each pose.

**Figure 10:**
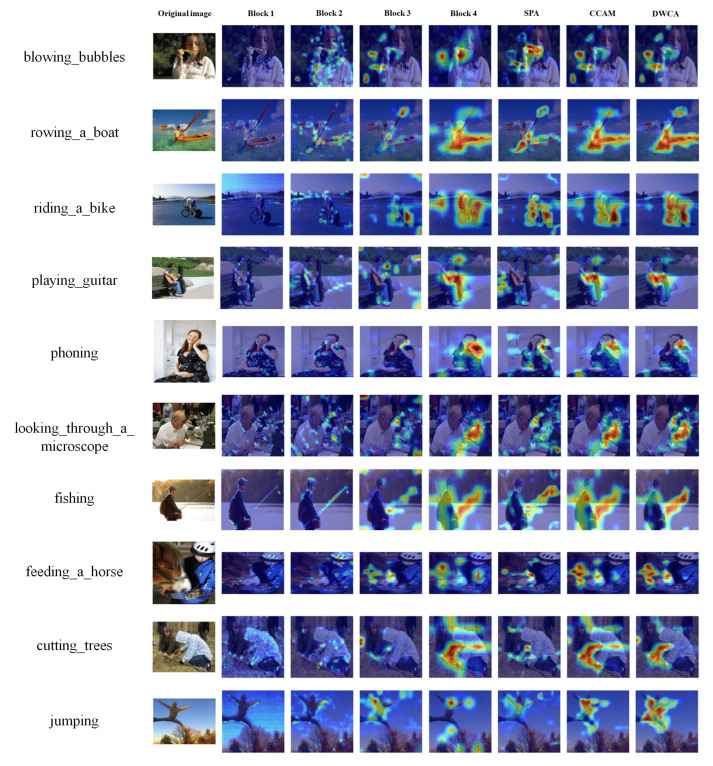
An illustration of Grad-CAM with the Stanford 40 Actions dataset. Bolder colors such as red represents stronger attentions rather than blue and lighter colors.

In Figure 10, which illustrates Grad-CAM on a standard dataset, the maps demonstrate how attention evolves from the earliest convolutional stages to the application of specialized attention modules. In Block 1, activations are generally dispersed, often covering large portions of the scene, including irrelevant background areas. Block 2 begins to capture some coarse action-related cues but still lacks clear separation between the object of interest and surrounding regions. By Block 3 and Block 4, the focus becomes more refined, with activations concentrating on areas such as the face and bubbles in blowing bubbles, the rider and bicycle frame in riding a bike, or the fisherman’s torso and fishing rod in fishing. The attention modules, such as SPA, CCAM, and DWCA, further enhance this focus by suppressing less relevant regions and sharpening key features. SPA often highlights the general action region, CCAM tends to capture multiple discriminative points across the subject, and DWCA frequently provides the most precise localization, as seen in its ability to lock onto the bubble wand, the body of the guitar, or the microscope eyepiece.

## 5. Conclusions

In this work, we presented a lightweight and modular attention-based architecture for human pose classification, leveraging a Swin Transformer backbone for efficient multi-scale feature extraction. By incorporating Spatial Attention, the Context-Aware Channel Attention Module, and our proposed Dual Weighted Cross Attention module, the model effectively fuses spatial and channel-wise cues while adaptively balancing attention-refined features with direct feature concatenations. Extensive experiments on the Yoga-82 dataset (in both main-class and subclass settings) and the Stanford 40 Actions dataset demonstrated that our approach consistently outperforms state-of-the-art baselines across multiple evaluation metrics, achieving high accuracy with only 0.79 million parameters. The integration of explainable AI techniques further enhances the interpretability and trustworthiness of the model’s predictions. Although our proposed method achieves strong performance, several challenges remain in yoga pose classification and related human pose recognition tasks. First, the high inter-class similarity between certain poses can still lead to misclassifications, especially when differences are subtle and localized to small body regions. Second, dataset noise and label inaccuracies, such as mislabeled or irrelevant images, can hinder generalization. Third, the current approach operates on static images, and therefore, cannot leverage the temporal information present in pose sequences. To address these issues, future work could explore incorporating part-based pose refinement modules to focus more precisely on discriminative body regions, applying automated data-cleaning pipelines to reduce noise, and integrating temporal modeling (e.g., Transformer-based video modules) to capture motion cues in sequential data.

## Figures and Tables

**Table 1:** Comparison between our proposed method and some state-of-the-art architectures on the Yoga-82 dataset. Numbers in bold represent the best performance.

Model	Number of Classes	Accuracy (%)	Precision (%)	Recall (%)	F1-Score (%)	Params (Million)
Verma, Manisha, et al. [32]	6	87.2	-	-	-	22.59
Borthakur, Debanjan, et al. [34]	6	85.0	-	-	-	-
Akash et al. [27]	6	85	87	83	83	-
Proposed method	6	**90.40**	**90.29**	**89.24**	**89.73**	**0.79**
Verma, Manisha, et al. [32]	20	84.42	-	-	-	22.59
Proposed method	20	**87.44**	**87.26**	**85.75**	**86.39**	**0.79**
Verma, Manisha, et al. [32]	82	78.88	-	-	-	22.59
Proposed method	82	**80.16**	**81.01**	**77.78**	**78.41**	**0.79**

**Table 2:** Comparison of our proposed method with other state of the art on Stanford 40 Actions dataset. Numbers in bold represent the best performance.

Model	MAP (%)	Accuracy (%)	Precision (%)	Recall (%)	F1-Score (%)
R*CNN [37]	90.90	-	-	-	-
ResNet-50 [38]	87.20	-	-	-	-
SAAM-Nets [39]	93.00	-	-	-	-
Multi-Branch Attention [40]	90.70	-	-	-	-
Top-down + Bottom-up Attention [41]	91.00	-	-	-	-
Multi-Attention Guided Network [35]	94.20	-	-	-	-
Body Structure Cues [36]	93.80	-	-	-	-
Hosseyni et al. [42]	93.10	-	-	-	-
Proposed method	**94.28**	**90.27**	**89.65**	**89.47**	**89.30**

**Table 3:** Comparison between different models chosen as our backbone. Numbers in bold represent the best performance.

Method	Accuracy (%)	Precision (%)	Recall (%)	F1-Score (%)
Resnet 50	79.88	79.13	78.00	78.38
VGG 16	65.70	64.41	61.93	62.21
Efficient Net	70.01	67.98	66.01	66.98
Swin transformer	**87.44**	**87.26**	**85.75**	**86.39**

**Table 4:** The effect of each module on our proposed method. Numbers in bold represent the best performance.

Baseline	Multi-Scale	Spatial Attention	CCAM	DWCA	Accuracy (%)	Precision (%)	Recall (%)	F1-Score (%)	Parameters (Million)	Flops (G)	Inference Time (ms)
Swin Transformer					64.50	62.65	61.53	61.74	0.14	15.17	20.07
	✓				77.69	79.10	73.39	74.62	0.25	15.26	19.87
		✓			70.88	71.26	65.75	66.72	0.14	15.17	19.69
			✓		69.51	69.47	63.19	64.54	0.14	15.17	20.99
				✓	70.76	69.69	66.24	66.34	0.60	16.61	25.7
	✓	✓			81.35	81.04	79.62	79.87	0.25	15.26	21.69
	✓		✓		82.19	81.71	81.14	81.02	0.25	15.26	21.61
	✓			✓	80.00	80.08	76.49	77.26	0.71	16.70	26.41
	✓	✓	✓		83.48	82.81	82.21	82.10	0.29	15.47	21.16
	✓		✓	✓	79.43	80.15	74.78	76.29	0.71	16.70	26.56
	✓	✓		✓	81.11	81.57	77.45	78.85	0.71	16.70	26.34
		✓	✓		72.20	71.33	67.99	68.69	0.14	15.17	20.05
		✓		✓	69.54	67.69	65.14	65.49	0.60	16.61	25.15
		✓	✓	✓	76.49	75.75	73.34	74.16	0.60	16.61	26.41
			✓	✓	70.25	69.35	63.93	64.45	0.60	16.61	25.88
	✓	✓	✓	✓	**87.44**	**87.26**	**85.75**	**86.39**	0.79	16.91	26.91

**Table 5:** The effect of different spatial dimensions and channel numbers on enhancing our method in CRSI. Numbers in bold represent the best performance.

Channels	Height	Width	Accuracy (%)	Precision (%)	Recall (%)	F1-Score (%)
64	56	56	78.47	79.34	74.54	75.69
64	28	28	78.53	78.52	74.23	75.42
64	14	14	80.06	79.19	76.45	77.44
64	7	7	79.49	79.84	74.68	75.95
128	56	56	**87.44**	**87.26**	**85.75**	**86.39**
128	28	28	80.18	79.51	76.71	76.89
128	14	14	78.29	77.65	75.85	76.22
128	7	7	82.52	82.47	79.56	80.41
256	56	56	78.59	78.63	76.12	76.85
256	28	28	77.21	79.01	72.68	74.31
256	14	14	79.43	77.73	77.08	76.92
256	7	7	77.78	78.24	73.12	74.43

**Table 6:** Comparison of different used convolution of our SPA and the spatial attention of CBAM. Numbers in bold represent the best performance.

Model	Conv 7 × 7	Conv 3 × 3	Accuracy (%)	Precision (%)	Recall (%)	F1-Score (%)
Spatial attention (from original cbam)	✓		80.06	79.64	77.32	78.01
Spatial attention (from original cbam)		✓	80.72	80.14	79.56	79.44
Our Spatial attention	✓	✓	**87.44**	**87.26**	**85.75**	**86.39**

**Table 7:** An ablation study to show the effect of using CCAM on our proposed method. Numbers in bold represent the best performance.

Model	Residual Connection	Conv 1 × 1	Accuracy (%)	Precision (%)	Recall (%)	F1-Score (%)
Non-Context-Aware Channel Attention		✓	82.07	82.68	78.87	80.24
Context-Aware Channel Attention	✓	✓	**87.44**	**87.26**	**85.75**	**86.39**

**Table 8:** An ablation study to show the effect of using DWCAA on our proposed method rather than other fusion strategies. Numbers in bold represent the best performance.

Model	Method	Accuracy (%)	Precision (%)	Recall (%)	F1-Score (%)
Proposed Method	Concat	83.48	82.79	82.74	82.57
Proposed Method	Addition	82.52	82.32	81.01	81.07
Proposed Method	Cross-Attention	85.10	85.64	82.88	84.10
Proposed Method	DWCA	**87.44**	**87.26**	**85.75**	**86.39**

## Data Availability

Both the Yoga-82 and Stanford 40 Actions datasets are publicly released for non-commercial research purposes under the terms provided by their original authors. All images used in this work are drawn directly from these datasets, and no additional personal data were collected. The dataset licenses and usage terms can be found at the respective repositories: Yoga-82: https://www.kaggle.com/datasets/akashrayhan/yoga-82 (accessed on 25 May 2025); Stanford 40 Actions: http://vision.stanford.edu/Datasets/40actions.html (accessed on 25 May 2025). Also the source code of this study is publicly available at https://github.com/alirezasa7/human-pose-and-action-classification (accessed on 25 May 2025).
